# Impact of the Addition of Baricitinib to Standard of Care Including Tocilizumab and Corticosteroids on Mortality and Safety in Severe COVID-19

**DOI:** 10.3389/fmed.2021.749657

**Published:** 2021-11-08

**Authors:** Mar Masiá, Sergio Padilla, José Alberto García, Javier García-Abellán, Andrés Navarro, Lucía Guillén, Guillermo Telenti, Paula Mascarell, Ángela Botella, Félix Gutiérrez

**Affiliations:** ^1^Infectious Diseases Unit, Hospital General Universitario de Elche, Universidad Miguel Hernández, Elche, Spain; ^2^Infectious Diseases Unit, Hospital General Universitario de Elche, Elche, Spain; ^3^Department of Clinical Pharmacy, Hospital General Universitario de Elche, Elche, Spain

**Keywords:** baricitinib, mortality, thrombosis, coinfection, COVID-19, SARS-CoV-2, tocilizumab, corticosteroids

## Abstract

**Background:** Baricitinib is a Janus kinase (JAK) inhibitor with a broader anti-inflammatory activity than tocilizumab and an antiviral potential although no head-to-head trials are available. The benefits of adding baricitinib to patients with COVID-19 experiencing clinical progression despite the standard of care (SOC), including corticosteroids and tocilizumab, are also unknown.

**Methods:** A cohort study included microbiologically confirmed COVID-19 hospitalizations. The primary outcome was 28-day mortality. Secondary outcomes were 60- and 90-day mortality, the composite outcome “28-day invasive mechanical ventilation (IMV) or death” and the safety of the combination. Propensity score (PS) matching was used to identify the association between baricitinib use and the outcomes of interest.

**Results:** Of 1,709 admissions, 994 patients received corticosteroids and tocilizumab and 110 of them received baricitinib after tocilizumab. PS matched 190 (95:95) patients with baricitinib + SOC vs. SOC, of whom 69.5% received remdesivir. No significant effect of baricitinib was observed on 28-day [39 events; adjusted hazard ratio (aHR), 0.76; 95% CI, 0.31–1.86], 60-day (49 events, aHR, 1.17; 95% CI, 0.55–2.52), or 90-day mortality (49 events; aHR, 1.14; 95% CI, 0.53–2.47), or on the composite outcome 28-day IMV/death (aHR, 0.88; 95% CI, 0.45–1.72). Secondary infections during hospitalization were not different between groups (17.9 vs. 10.5%, respectively; *p* = 0.212) and thromboembolic events were higher with baricitinib (11.6% vs. 3.2%; *p* = 0.048), but differences vanished after the adjustment [aHR 1.89 (0.31–11.57), *p* = 0.490].

**Conclusion:** The addition of baricitinib did not substantially reduce mortality in hospitalized patients with COVID-19 having clinical progression despite the therapy with tocilizumab and corticosteroids. The combination of baricitinib and tocilizumab was not associated with an increased risk of secondary infections or thromboembolic events.

## Introduction

Severe COVID-19 is characterized by an imbalanced host response to SARS-CoV-2 infection, resulting in cytokine dysregulation and wide-ranging immuno-inflammatory derangements leading to lung damage and multi-organ dysfunction (1). Therapeutic strategies in this setting are based on a combination of antiviral and primarily immunomodulatory therapy although there is no consensus on the optimal regimen composition (2, 3) and mortality remains unacceptably high (4). Among immune modulators, dexamethasone has been shown to reduce mortality (5) and is currently considered the mainstay of treatment for severe and critical illness (2, 3). Tocilizumab, an interleukin-6 (IL-6) receptor antagonist, improved the survival in hospitalized patients with hypoxia and systemic inflammation and critically ill patients (6–8), and was incorporated into treatment guidelines for severe non-critical and critical disease (3). More recently, the inhibitors of the Janus kinase (JAK) family have also been associated with a decreased risk of mortality (9–11). Baricitinib is a JAK1/2 inhibitor that has emerged as an alternative therapeutic option, mainly for patients requiring high-flow oxygen or non-invasive ventilation (NIV), in whom the drug has shown the most pronounced benefit in reducing mortality and accelerating improvement (2, 3, 9, 10, 12). The mechanism of action of baricitinib includes theoretical advantages over tocilizumab, as baricitinib decreases the concentration of several cytokines and inflammatory mediators involved in the pathogenesis of COVID-19 in addition to IL-6 and has an additional potential antiviral activity (13).

Clinical experience with baricitinib in patients with COVID-19 is limited compared to tocilizumab. To date, no head-to-head trials are available to assess the best anti-cytokine option for patients with severe disease. Furthermore, it is not unusual that patients initially receiving tocilizumab progress to requiring high-flow oxygen, and it is unclear whether sequential therapy with baricitinib could offer additional benefits. A drawback against their combination would be a potential increased risk of severe infections or thrombosis.

We evaluated the impact of baricitinib on the mortality and safety of hospitalized patients with severe COVID-19 and clinical progression despite the therapy with the standard of care (SOC), including tocilizumab and corticosteroids.

## Methods

A longitudinal prospective study was conducted in a cohort of patients hospitalized for COVID-19 between March 10, 2020, and April 30, 2021. Criteria for hospital admission were SARS-CoV-2 infection confirmed trough real time PCR (RT-PCR), abnormal findings on chest x-ray, and/or severity criteria, including oxygen saturation < 94% and CURB-65 ≥ 2. Eligibility criteria for this study included the therapy with corticosteroids during admission and, for patients receiving baricitinib, concomitant therapy with tocilizumab.

Patients were managed according to a predefined diagnostic and therapeutic local protocol approved by the COVID-19 Institutional Committee of Hospital General Universitario de Elche (14). This protocol consisted of the standardized collection of clinical variables and serial blood and nasopharyngeal sampling obtained on admission and every 48 h during the hospital stay for SARS-CoV-2 PCR, serological and laboratory measurements, including the levels of IL-6, ferritin, C-reactive protein (CRP), D-dimer, and neutrophil-to-lymphocyte ratio (NLR). The therapy for COVID-19 was given following regularly updated institutional guidelines. Patients received antimicrobial and/or immunomodulatory therapy containing lopinavir/ritonavir, hydroxychloroquine, azithromycin, interferon-β-1b, or remdesivir ± methylprednisolone (125–250 mg as an intravenous bolus) ± tocilizumab during the first wave (*n* = 39/994 patients), and dexamethasone (6 mg daily) ± remdesivir ± tocilizumab according to pre-specified criteria (11) during the second wave and third wave (*n* = 858/994 patients). From December 2020, after the Food and Drugs Administration (FDA) release of an emergency use authorization of baricitinib (15), the drug was added to therapy when patients required high-flow nasal oxygen or NIV after obtaining verbal informed consent. Exclusion criteria for baricitinib use included creatinine clearance < 30 ml/min, absolute lymphocyte count < 0.5 × 10^9^ cells/L, absolute neutrophil count < 1 × 10^9^ cells/L, hemoglobin < 8 g/dl, pregnancy, and the suspicion of bacterial infection as the cause of clinical deterioration.

In addition, antibiotic therapy with azithromycin and ceftriaxone was added when clinical or laboratory data suggested bacterial coinfection, including lobar pulmonary infiltrate of chest x-ray, procalcitonin > 0.5 ng/ml, purulent or hemoptoic sputum or significant bacterial isolation, or positive pneumococcal urinary antigen. All patients received antithrombotic prophylaxis (16) with enoxaparin, 40/60 mg/day subcutaneous (sc) dependent on weight, or 1 mg/kg if the risk factors, including D-dimer > 1.5 μg/L, IL-6 > 40 pg/ml, lymphocytes < 800 × 10^9^ × L, or ferritin ≥1,000 μg/L, were present.

Patients were followed up at months 1, 2, 3, 6, and 12 after discharge. On each visit, their blood samples were taken for routine laboratory tests and biomarkers, a nasopharyngeal swab for SARS-CoV-2 PCR, chest x-ray, and plasma aliquots were drawn and frozen, and they filled a questionnaire.

The main outcome variable was all-cause 28-day mortality. Secondary outcome variables were 60- and 90-day mortality and a composite variable that included 28-day mechanical ventilation or death. The risk of secondary infections and thromboembolic events was also analyzed as secondary safety outcomes. Secondary infections were defined by the clinical diagnosis of an infection occurring after a follow-up initiation of baricitinib and tocilizumab, confirmed with microbial tests.

### Statistical Analyses

Descriptive statistics were used to summarize the baseline characteristics of the cohort. Continuous data were reported as medians ± 25th and 75th percentiles (Q1, Q3), and count data were presented along with percentages. Wilcoxon or Student's *t*-test was used to compare continuous variables, and the chi-squared or Fisher's exact test was used for a categorical variable comparison.

Main analyses were based on time-to-event methods. The follow-up of patients for this analysis started the day of baricitinib initiation for the tocilizumab and baricitinib group, and the day from high-flow oxygen requirement for the tocilizumab group.

Multivariate propensity score (PS) matching the logistic regression model was fitted with a 1:1 ratio among groups to compare patients receiving baricitinib + tocilizumab with patients receiving tocilizumab without baricitinib who required high-flow oxygen through nasal cannula or NIV. Matching variables included the relevant baseline data that might have affected treatment decisions, including sex, age, Charlson comorbidity index, baseline fraction of inspired oxygen, WHO COVID-19 severity ordinal scale, Sequential Organ Failure Assessment (SOFA) score, and Chronic Kidney Disease Epidemiology Collaboration (CKD-EPI) estimation of glomerular filtration rate. Standardized mean differences (SMDs) were calculated to examine the balance of a covariate distribution between treatment groups. Because SMD is independent of the unit of measurement, it allows a comparison between variables with different units of measurement. Matched patients were compared for the primary and secondary outcome variables and the safety outcome risk of secondary infection or thromboembolism. To further adjust for the covariates that remained unbalanced between treatment groups after matching, adjusted Cox proportional hazard regression for binomial outcomes or Poisson regression for ordinal outcomes were run. To represent the temporal changes of the levels of the biomarkers, local polynomial regression models were employed using weighted least squares to estimate the performance of each biomarker according to the day of initiation of baricitinib or the day of high-flow oxygen requirement for the tocilizumab group. Differences in temporal trends between biomarkers were analyzed through linear mixed models in which an interaction term of the day of tocilizumab initiation and response was included. Statistical analyses were performed using R software (17).

## Results

The study population comprised 1,709 patients who were admitted between March 1, 2020, and March 31, 2021, to Hospital General Universitario de Elche, Spain. Of them, 994 (58.2%) had the information needed for being included in the final study cohort. The reasons for the withdrawal of patients were not receiving either tocilizumab or baricitinib (706 patients) and receiving baricitinib before or without tocilizumab (nine patients) (Supplementary Figure 1). Characteristics of the individuals not included in the analyses and their comparison with those included are shown in Supplementary Table 1.

Death from any cause from hospital admission through day 28 occurred in 74 (7.4%) patients, in 4% during the first wave and in 9.7% during the second wave, and 93 (9.3%) were admitted to the Intensive Care Unit (ICU) during the hospital stay. Non-invasive respiratory support strategies were required in 123 (12%) patients [high-flow nasal oxygen in 109 (11%); continuous positive airway pressure or bilevel NIV in 36 (4%)] and invasive mechanical ventilation (IMV) in 75 (7.5%) patients (Table 1). In 73 (97%) patients on IMV, inotropic support and/or renal replacement therapy techniques were also necessary. All the study patients received at least one dose of tocilizumab and corticosteroids, of whom 268 (27%) received high-dose corticosteroid pulses and 713 (71.7%) received antiviral therapy with remdesivir.

**Table 1:** Propensity score (PS) analysis.

**Variable**	**Before propensity score matching**			**After propensity score matching**	
	**All**	**Tocilizumab + Baricitinib**	**Tocilizumab**	**Tocilizumab + Baricitinib**	**Tocilizumab**
Patients, no.	994	110	884	95	95
Male sex	636 (64)	74 (67)	562 (64)	60 (63)	56 (59)
Age, years	66 (54, 76)	72 (60, 79)	65 (54, 76)	72 (62, 78)	72 (60, 80)
CCI, points	3 (1, 4)	3 (2, 5)	2 (1, 4)	3 (2, 5)	4 (1, 6)
Any comorbidity^*^	735 (74)	99 (90)	636 (72)	85 (90)	76 (80)
Cardiovascular disease	311 (31)	47 (43)	264 (30)	41 (43)	40 (42)
Hypertension	465 (47)	64 (58)	401 (45)	53 (56)	55 (58)
Diabetes	236 (24)	35 (32)	201 (23)	31 (33)	35 (37)
Chronic obstructive lung disease	54 (5)	12 (11)	42 (5)	12 (13)	7 (7)
WHO COVID-19 severity score	4 (4, 4)	4 (4, 6)	4 (4, 4)	4 (4, 6)	4 (4, 5)
SOFA score	2 (1, 3)	3 (2, 4)	2 (1, 2)	3 (2, 4)	3 (2, 3)
Peak FIO2, %	36 (32, 50)	100 (100, 100)	36 (32, 50)	100 (91, 100)	100 (90, 100)
Remdesivir use	713 (72)	86 (78)	627 (71)	72 (76)	60 (63)
Baricitinib use	110 (11)	110 (100)	0 (0)	95 (100)	0 (0)
eGFR, ml/min	89 (69, 103)	83 (55, 98)	90 (73, 104)	77 (54, 99)	77 (50, 95)
eGFR ≤ 30 ml/min	50 (5)	9 (8)	41 (5)	8 (8)	14 (15)
CRP, mg/L	61 (20, 118)	33 (6, 104)	81 (50, 128)	33 (6, 105)	81 (50,129)
IL-6, pg/mL	180 (74, 447)	196 (84, 485)	167 (44, 439)	196 (84, 486)	167 (44, 439)
D-dimer, mcg/mL	1.0 (0.6, 2.0)	1.1 (0.6, 2.1)	0.9 (0.5, 1.8)	1.1 (0.6, 2.1)	0.9 (0.5, 1.8)
Bilateral lung infiltrates	882 (89)	107 (97)	775 (88)	92 (97)	87 (92)
Length of symptoms at admission, days	6 (3, 9)	5 (3, 8)	6 (3, 9)	5 (3, 8)	6 (3, 9)
Length of hospital stay, days	6 (4, 11)	19 (13, 27)	6 (4, 9)	18 (12, 26)	11 (7, 19)
**Clinical events at 28 days**					
Overall mortality	74 (7)	28 (26)	46 (5)	24 (25)	15 (16)
NIV or HFO	123 (12)	94 (85)	29 (3)	80 (84)	19 (20)
ICU admission	93 (9)	42 (38)	51 (6)	34 (36)	18 (19)
Mechanical ventilation	75 (8)	34 (31)	41 (5)	26 (27)	13 (14)
Documented infection	49 (5)	19 (17)	30 (3)	17 (18)	10 (11)
Thromboembolic events	48 (5)	21 (21)	27 (3)	11 (12)	3 (3)
Overall mortality at 60 days	90 (9)	33 (30)	57 (6)	29 (31)	20 (21)
Overall mortality at 90 days	91 (9)	33 (30)	58 (7)	29 (31)	20 (21)

*The characteristics of both populations of patients, before and after PS matching, and unadjusted and adjusted hazard ratios (aHRs) for 28-day all-cause mortality between the mated groups are shown*.

* *This category included at least one of the following: diabetes, cardiovascular (including hypertension) respiratory, kidney, neurological disease, cirrhosis, or malignant neoplasm. Summary statistics are provided as medians with Q1, Q3, or numbers with percentages as appropriate*.

*CCI, Charlson comorbidity index; WHO, World Health Organization; SOFA, Sequential Organ Failure Assessment; FiO2, baseline fraction of inspired oxygen; eGFR, Chronic Kidney Disease Epidemiology Collaboration (CKD-EPI) estimation of glomerular filtration rate; CRP, C-reactive protein; IL, Interleukin; NIV, Non-invasive respiratory support; HFO, high-flow nasal oxygen; ICU, Intensive Care Unit*.

A total of 119 (6.6%) patients were treated with baricitinib, of whom 110 received the drug after tocilizumab initiation. Baricitinib was initiated after a median (Q1–Q3) of 3 (1–6) days from admission, the median treatment duration was 14 days, and 104 (96%) patients completed at least 14 days of therapy. In addition to previous corticosteroids and tocilizumab administration, 78% of the patients with baricitinib also received antiviral therapy with remdesivir, and 80% with high-dose corticosteroid pulses.

### PS-Matched Patients

After 1:1 PS matching, 95 patients in each group (baricitinib and control groups) were compared for the primary outcome and secondary outcomes. Their baseline characteristics according to the study group before and after PS matching are presented in Table 1. Absolute standardized mean (ASM) differences between the two study groups diminished compared to those previous to propensity matching, and values of *p* for ASM tests were above 0.05, broadly reflecting the adequate balance between the two groups. Although the difference in hospital length of stay between groups decreased after PS matching, it remained significantly longer in the baricitinib group [median (Q1, Q3); 18 (12, 26) vs. 11 (7, 19) days; *p* = 0.001]. In the adjusted Poisson regression analysis, baricitinib use was not associated with a significantly different hospital stay [risk ratio (95% CI) 1.05 (0.96–1.14); *p* = 0.304].

**Figure 1:**
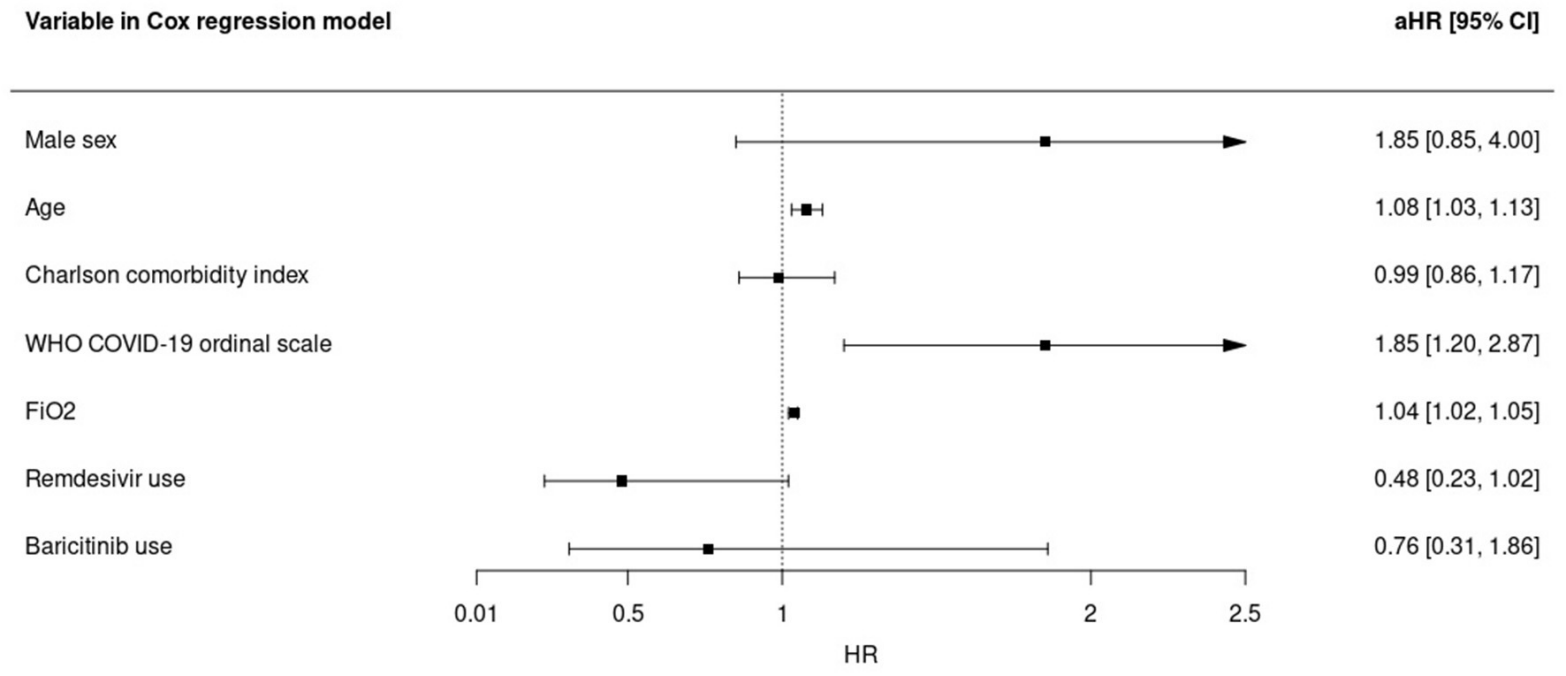
Predictors for overall 28-day mortality in the multivariate Cox regression model in subjects receiving tocilizumab. aHR, adjusted hazard ratio; WHO, World Health Organization; FiO2, time-varying fraction of inspired oxygen.

All-cause 28-day mortality rate in baricitinib recipients was 25.2% (24 deaths) compared with 15.7% (15 deaths) in matched controls, with a non-significant difference in the adjusted time-to-death analysis [adjusted hazard ratio (aHR), 0.76; 95% CI, 0.31–1.86]. Time-varying FiO2 (aHR, 1.04; 95% CI, 1.02–1.05; per percentage unit increase), age (aHR, 1.08; 95% CI, 1.03–1.13; per year increase), and WHO ordinal COVID-19 scale category (aHR, 1.85; 95% CI, 1.20–2.87 per category increase) were also associated with mortality in a multivariate analysis. In contrast, remdesivir use showed a trend to lower mortality risk (aHR, 0.48; 95% CI, 0.23–1.02) (Figure 1). In a sensitivity analysis using the severity NIAID ordinal scale instead of the WHO ordinal scale, the results were similar (Supplementary Figure 3).

**Figure 2:**
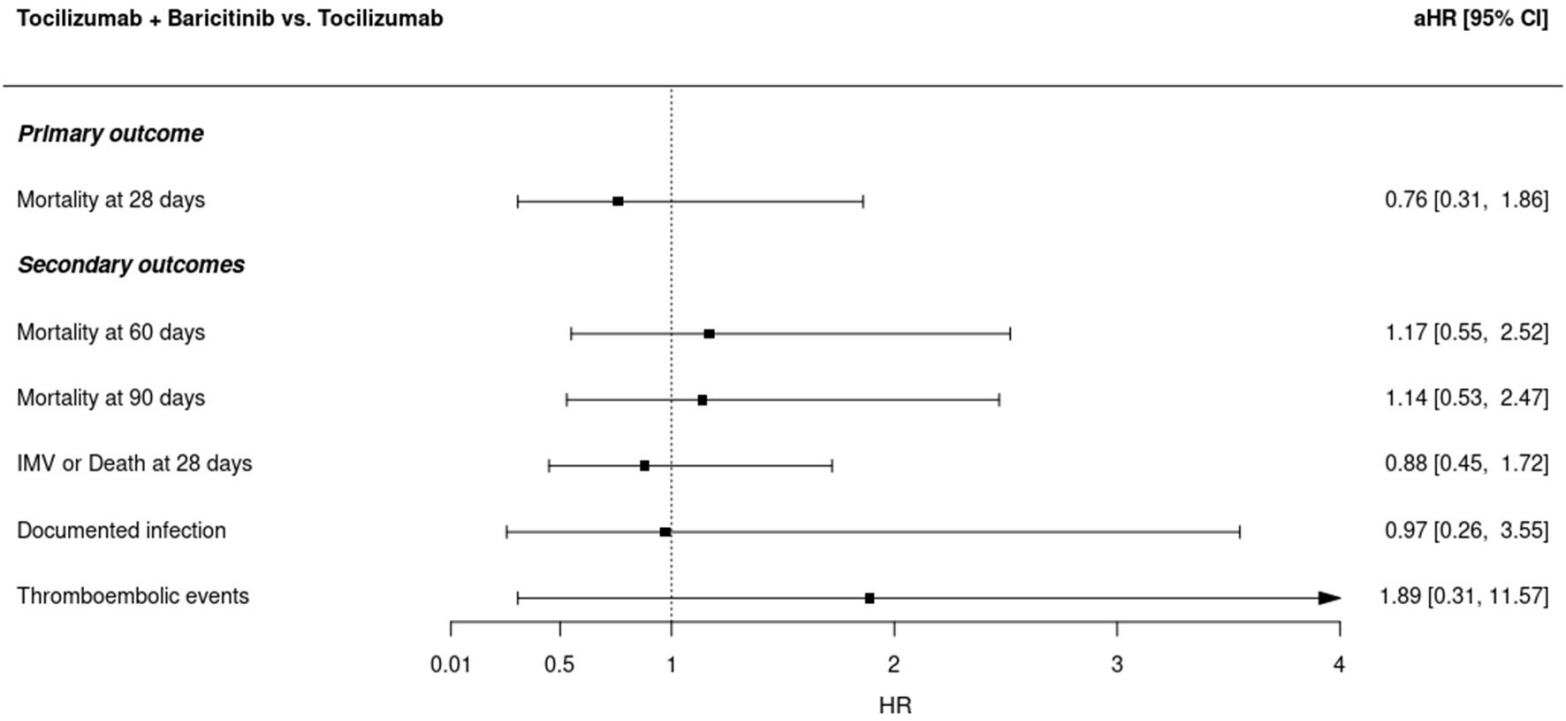
Adjusted Cox regression model hazard ratios for the combination of tocilizumab plus baricitinib vs. tocilizumab alone in different study outcomes. Cox regression models were adjusted by sex, age, Charlson comorbidity index, WHO COVID-19 severity ordinal scale, the time-variant fraction of inspired oxygen, and remdesivir use. aHR, adjusted hazard ratio; IMV, invasive mechanical ventilation.

Secondary endpoint analyses did neither identify a significant effect of baricitinib. All-cause 60- (aHR, 1.17; 95% CI, 0.55–2.52) and 90-day (aHR, 1.14; 95% CI, 0.53–2.47) mortality or time to the composite outcome 28-day mechanical ventilation or death (aHR, 0.88; 95% CI, 0.45–1.72) were not different between groups in the adjusted analyses (Figure 2). Sensitivity analysis of the PS with a caliper width of 0.2 of the SD of the logit for the 1:1 matching ratio showed similar results (Supplementary Table 2).

The incidence of secondary coinfections during the COVID-19 hospitalization did not differ between patients receiving baricitinib and the control group [17 (17.9%) vs. 10 (10.5%); *p* = 0.212; aHR 0.97 (0.26–3.55)]. Supplementary Table 3 shows the secondary infections occurring during the study period. There was a higher number of thromboembolic events (pulmonary thromboembolism and deep venous thrombosis) during hospital admission of patients receiving baricitinib [11 (11.6%) vs. 3 (3.2%); *p* = 0.048] in the unadjusted analysis. However, after Cox's additional adjustment for age, sex, time-varying FiO2, Charlson comorbidity score, WHO COVID-19 ordinal scale, and remdesivir use, the risk was not different between treatment groups [aHR 1.89 (0.31–11.57), *p* = 0.490] (Supplementary Figure 2).

### Viral RNA Shedding and Biomarker Changes

**Figure 3:**
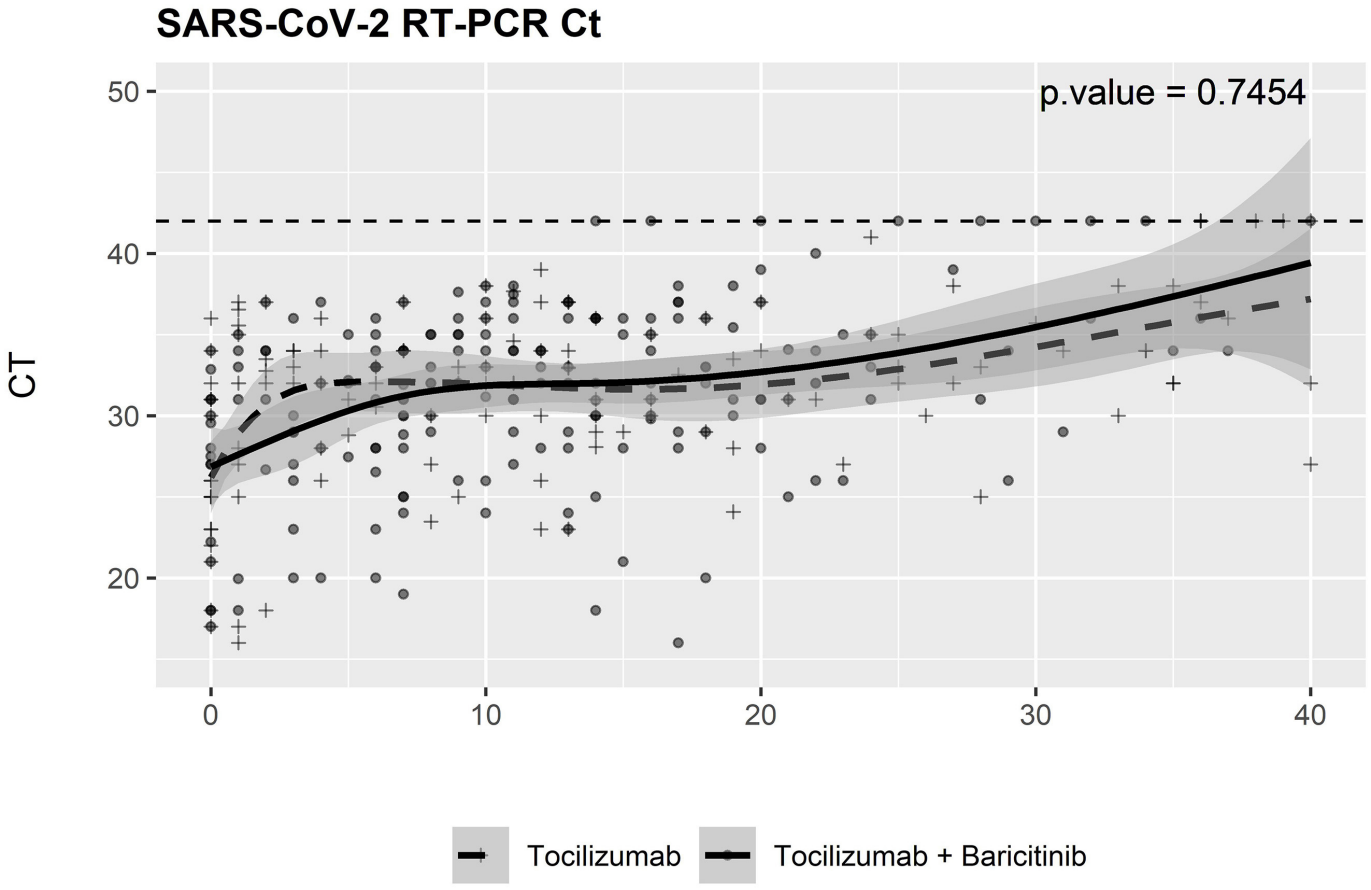
Severe acute respiratory syndrome coronavirus 2 (SARS-CoV-2) virological changes during a follow-up. SARS-CoV-2 RT-PCR test cycle threshold results were obtained from baseline. RT-PCR, reverse transcriptase-PCR; Ct, cycle threshold.

There were no significant differences in the dynamics of SARS-CoV-2 RT-PCR cycle thresholds obtained from nasopharyngeal swabs between patients receiving combined therapy with baricitinib and tocilizumab and the control group (Figure 3).

**Figure 4:**
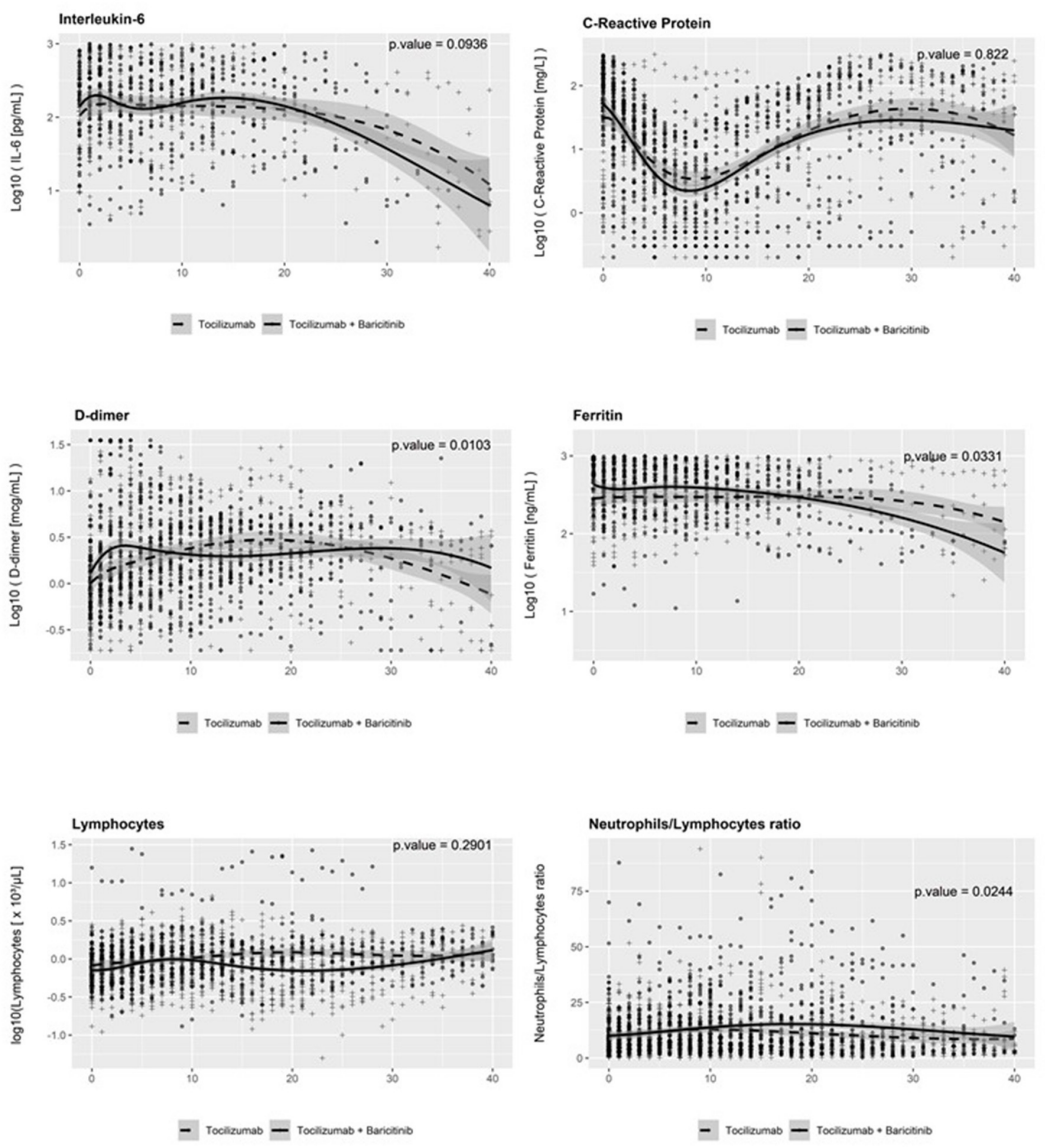
Temporal changes in serum levels of biomarkers from baseline. IL-6, interleukin-6.

At baseline, the plasma levels of biomarkers analyzed (inflammation: CRP, ferritin, and IL-6; fibrinolysis: D-dimer) were similar, with the exception of ferritin, which showed higher concentrations in patients receiving baricitinib + tocilizumab. Thereafter, all of them showed a decreasing trend with minor differences in the trajectories between treatment groups and initial small and transient increases in the levels of IL-6 and D-dimer (Figure 4).

## Discussion

In this cohort study, baricitinib did not show a benefit on mortality when added to SOC therapy, including tocilizumab, corticosteroids, and frequently remdesivir, in hospitalized patients with COVID-19 having clinical progression requiring oxygen delivery through a high-flow device or NIV. No increased incidence of infection or thrombotic phenomena were observed when tocilizumab and baricitinib were sequentially used.

Interleukin-6 receptor antagonists and JAK inhibitors are the immune modulators that, along with corticosteroids, have demonstrated a benefit on the survival in patients with severe COVID-19 (9–11). IL-6 has been shown to play a central role in the immune dysregulation that characterizes COVID-19 pneumonia, in which the levels of this cytokine have been associated with disease severity (18). Tocilizumab is a monoclonal antibody that competitively inhibits IL-6 binding to its receptor, thereby blocking IL-6 signaling and reducing inflammation (6). Baricitinib has a broader anti-cytokine activity than tocilizumab as it has been shown to *in vitro* decrease SARS-COV-2-specific response mediated by IFN-γ, IL-17, IL-1β, IL-6, TNF-α, IL-4, IL-13, IL-1ra, IL-10, GM-CSF, FGF, IP-10, MCP-1, MIP-1β (13, 19), and the general immune response through a rapid recovery of circulating T- and B-cell frequencies (20). In addition, baricitinib also acts as a potential antiviral drug through the inhibition of clathrin-mediated endocytosis of SARS-CoV-2 into cells (21). In light of this, a sequential combination of baricitinib after tocilizumab therapy could be expected to have an additional beneficial impact. However, we found no advantage of JAK inhibition after IL-6 receptor blockade on the survival of patients. This may suggest a preponderant role of IL-6 over other cytokines in the pathogenesis and outcome of severe COVID-19 (22). Tocilizumab has a prolonged half-life of 11–13 days (23), and the effects on IL-6 are likely to persist when baricitinb is administered. Moreover, the inhibitory effects of baricitinib on interferon-gamma, which has a broad-spectrum antiviral activity at multiple stages (24), might result in a hypothetical negative impact when added to the analogous effects in the defense against viral infections of IL-6 blockade. Another factor potentially contributing to the absence of a benefit with baricitinib could be related to the concurrent therapy with corticosteroids, which have also been shown to increase survival, although baricitinib demonstrated in a randomized trial a reduction in mortality against placebo in patients mostly on corticosteroid therapy (10). While we found no benefit of the addition of baricitinib to a regimen containing tocilizumab and dexamethasone, our study does not allow comparing the effect of baricitinib with that of tocilizumab or dexamethasone in patients with severe COVID-19, as all patients received the SOC including tocilizumab and dexamethasone.

Our study has analyzed the influence of the combination of baricitinib and tocilizumab on viral clearance and found no differences in the trajectory of RT-PCR cycle threshold in comparison with that of tocilizumab as a sole anticytokine agent. Although baricitinib has a potential antiviral effect, the majority of patients in our cohort were also receiving remdesivir, an antiviral drug with demonstrated benefits in COVID-19 (25–27), which could overshadow the effect of baricitinib. Remdesivir use showed a trend to increased survival in our study. Although the use of remdesivir was slightly more frequent in patients receiving baricitinib, the difference was far from statistical significance.

One of the uncertainties raised with the combination of baricitinib and tocilizumab is the hypothetical additive risk of infection (2). Although no contraindication exists for the concomitant use of both drugs, the potential increased risk of infections had been warned, as both agents could mutually enhance their anticytokine activity, leading to impaired innate and adaptive immune responses to viral, parasitic, and bacterial infections (28, 29). Our patients were thoroughly monitored during their hospital stay, with lab tests every other day including biomarkers of infection, and an additional protocol for patients experiencing fever or clinical progression that included further diagnostic tests to exclude opportunistic infections and empirical antimicrobial therapy. While both agents have been linked with an increased risk of infections in patients with rheumatic disease (30–33), clinical trials, and meta-analyses with each drug have not verified a higher frequency of serious infections in patients with COVID-1 (9, 10, 34–36). We neither observed significant differences in the incidence of secondary infections with the combination of baricitinib plus tocilizumab compared to tocilizumab in our cohort even though all patients received additionally corticosteroids. The shorter duration of the immunomodulatory therapy in COVID-19 compared to rheumatic diseases, the frequent use of antibiotics in our cohort, and the fact that the comparator was tocilizumab plus corticosteroids might have contributed to minimizing differences between treatment groups.

We observed a higher incidence of thromboembolic events in patients receiving baricitinib in our study. However, the adjustment for disease severity and the factors associated with thrombosis risk did not confirm a greater risk. COVID-19 is associated with coagulopathy and increased frequency of thrombosis (37), and this was another concern regarding a combined therapy with baricitinib and tocilizumab. Moreover, both agents have been linked with a greater incidence of venous thromboembolism in patients with rheumatoid arthritis (30, 31, 38) although two recent meta-analyses of double-blinded randomized controlled trials with JAK-inhibiting therapies did not confirm an increased risk (39, 40). The analysis of the levels of D-dimer in our cohort did not show significant differences between the two treatment groups. Transient increases and decreases in D-dimer levels have been reported during the therapy with tocilizumab, and the same changes have also been described in patients with COVID-19 (41, 42).

The influence of baricitinib or tocilizumab on inflammation biomarkers had been assessed following the therapy with each drug (13, 14), but the added effects of baricitinib plus tocilizumab on inflammation had not been explored. There were no differences in the levels of neutrophils and lymphocytes between treatment groups and, despite minor differences in the trajectories IL-6, CRP, and ferritin, the magnitude of such differences did neither suggest an overt additive effect of the combination of the anti-cytokine agents on the inflammation biomarkers explored.

The observational nature of this study is a limitation, as residual confounding by baricitinib indication cannot be excluded despite adjustments. Actually, despite the PS matching, potential imbalances due to unmeasured comorbidities and comedications between the two study groups cannot be excluded. Nevertheless, a more exigent matching algorithm for the PS showed similar results. Another limitation is the sample size of this study, which implies that a significant difference between groups could not be excluded for a lower effect size of baricitinib. A strength of this study is the novelty of the information about the additive effect of baricitinib over tocilizumab in patients with disease progression, including the influence of the combination on viral shedding and inflammation/coagulation. This study also contributes to expanding the information about the effects of the triple combination of corticosteroids, tocilizumab, and remdesivir in patients with COVID-19, of which limited data exist to date.

In conclusion, when used in combination, baricitinib did not show additional benefits to tocilizumab, at least of great magnitude, on survival in our cohort of patients with severe COVID-19 undergoing SOC therapy, including corticosteroids and commonly remdesivir. Combined therapy was not associated with an increased risk of infection and probably not with thromboembolic events, although further studies are desirable to confirm our findings.

## Data Availability Statement

The raw data supporting the conclusions of this article will be made available by the authors, without undue reservation.

## Ethics Statement

The studies involving human participants were reviewed and approved by Ethics Committee of Hospital General Universitario de Elche (PI 46/2020). Written informed consent for participation was not required for this study in accordance with the national legislation and the institutional requirements.

## Author Contributions

MM: conceptualization, methodology, writing—original draft preparation, and supervision. SP: writing—original draft preparation, software, and formal analysis. JG: formal analysis and writing—review and editing. JG-A, LG, GT, PM, and ÁB: data curation and writing—review and editing. AN: investigation and writing—review and editing. FG: conceptualization, methodology, writing—original draft preparation, reviewing and editing, funding acquisition, and supervision. All authors contributed to the article and approved the submitted version.

## Funding

This study was supported by the RD16/0025/0038 project as a part of the Plan Nacional Research + Development + Innovation (R + D + I) and cofinanced by Instituto de Salud Carlos III - Subdirección General de Evaluación y Fondo Europeo de Desarrollo Regional; Instituto de Salud Carlos III [Fondo de Investigaciones Sanitarias (Grant Numbers: PI16/01740; PI18/01861; CM 19/00160; and COV20-00005)] and ILISABIO (A-32 2020).

## Conflict of Interest

The authors declare that the research was conducted in the absence of any commercial or financial relationships that could be construed as a potential conflict of interest.

## Publisher's Note

All claims expressed in this article are solely those of the authors and do not necessarily represent those of their affiliated organizations, or those of the publisher, the editors and the reviewers. Any product that may be evaluated in this article, or claim that may be made by its manufacturer, is not guaranteed or endorsed by the publisher.

## Members of the COVID-19-Elx Group

Félix Gutiérrez, Mar Masiá, Eduardo García, Antonia Mora, Sergio Padilla, Guillermo Telenti, Lucia Guillen, Cristina Bas, María Andreo, Fernando Lidón, Vladimir Ospino, Francisco López, Oscar Torregrosa, José López, Fernando Bonilla, Clara Escolano, Marta Fernández, Vanesa Agulló, Gabriel Estañ, Andrés Navarro, Lucía Zamora, Sandra Ruiz, Carlos Baeza, Antonio Ramón, Vicente Cantó, Vicente Boix, Ricardo Mora, Rafael Julio Simón, Irene Pascual, Javier García, Alejandro de la Torre, Cristina Martínez, Leticia Alonso, Joan Sanchís, Ángela Botella, Paula Mascarell, María Selene Falcón, Jose Carlos Asenjo, Carolina Ding, Luis Marhuenda, Enrique Valdeolivas, and Carolina Garrido.
