# Supplementary material for: Impact of the Addition of Baricitinib to Standard of Care Including Tocilizumab and Corticosteroids on Mortality and Safety in Severe COVID-19
Source: Front Med (Lausanne). 2021 Nov 8;8:749657. doi: 10.3389/fmed.2021.749657 (PMC8606519; doi:10.3389/fmed.2021.749657)
Supplement: Supplementary file 1 [file Data_Sheet_1.docx]

**Figure S-1. Flowchart of patients in the study.**


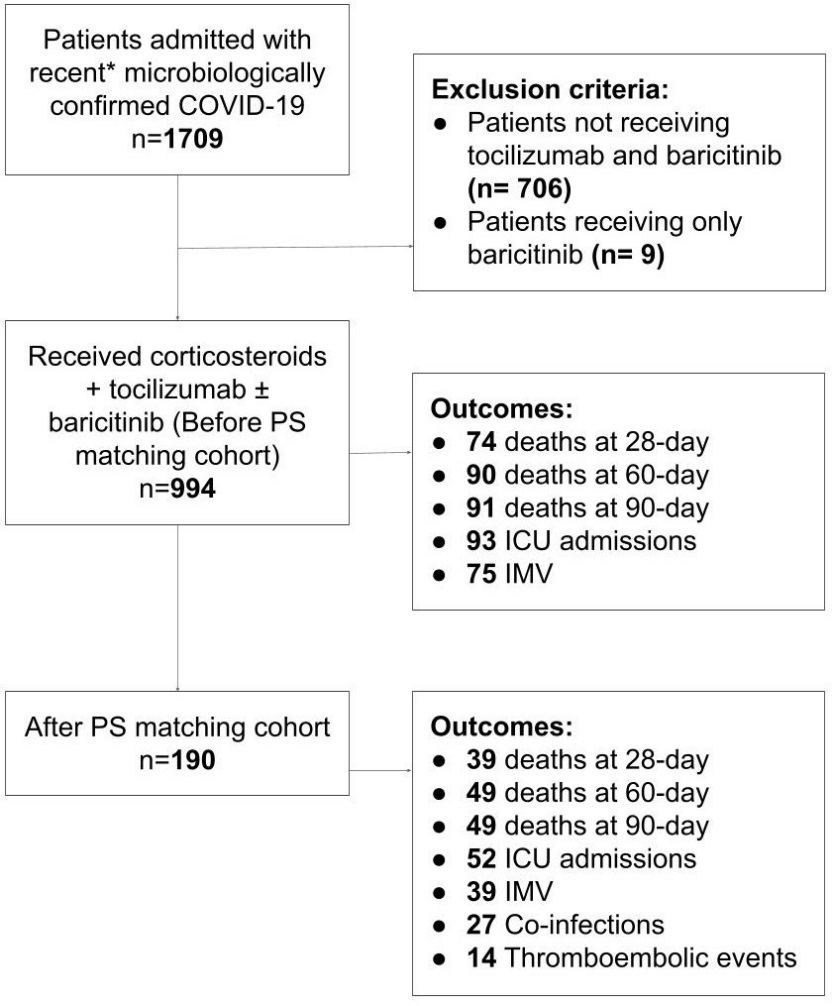


*A recent COVID-19 diagnosis was considered when the first ever positive patient’s SARS-CoV-2 RT-PCR was made less than 15 days prior to admission or during admission. Abbreviations: PS, propensity score; ICU, intensive care unit; IMV, invasive mechanical ventilation.

**Table S-1. Baseline characteristics of patients excluded because of missing information.**

| **Variable** |  |  | **Excluded**  **(n=715)** | **Included**  **(n=994)** | **p value** |
| --- | --- | --- | --- | --- | --- |
| **Male sex** |  |  | 344 (48) | 636 (64) | 0.001 |
| **Age, years** |  |  | 67 (54, 80) | 66 (54, 76) | 0.095 |
| **Any comorbidity***  **CCI, median (Q1, Q3) points**  **Cardiovascular disease**  **Hypertension**  **Diabetes**  **Chronic obstructive lung disease** |  |  | 507 (71)  3 (1, 5)  219 (31)  304 (43)  163 (23)  55 (8) | 735 (74)  3 (1, 4)  311 (31)  465 (47)  236 (24)  54 (5) | 0.169  0.015  0.791  0.085  0.685  0.071 |
| **WHO COVID-19 severity score**  **FIO2 on admission, %**  **Remdesivir use**  **Baricitinib use** |  |  | 4 (4, 4)  28 (21, 32)  188 (26)  9 (1) | 4 (4, 4)  28 (28, 35)  713 (72)  110 (11) | 0.069  0.001  0.001  0.001 |
| **eGFR, ml/min**  **CRP, mg/L**  **IL-6, pg/mL**  **D-dimer, mcg/mL**  **Length of hospital stay, days** |  |  | 88 (58, 104)  26 (10, 65)  18 (6, 41)  0.7 (0.4, 1.5)  5 (3, 10) | 89 (69, 103)  61 (20, 118)  180 (74, 447)  1.0 (0.6, 2.0)  6 (4, 11) | 0.170  0.001  0.001  0.491  0.001 |
| **Clinical events at 28 days**  **Overall mortality**  **ICU admission**  **Mechanical ventilation**  **Documented infection**  **Thromboembolic events**  **Overall mortality at 60 days**  **Overall mortality at 90 days** |  |  | 71 (10)  51 (7)  38 (5)  27 (4)  23 (4)  85 (12)  92 (13) | 74 (7)  93 (9)  75 (8)  49 (5)  48 (5)  90 (9)  91 (9) | 0.003  0.112  0.076  0.285  0.264  0.063  0.017 |
|  |  |  |  |  |  |

*This category included at least one of the following: diabetes, cardiovascular (including hypertension) respiratory, kidney, neurological disease, cirrhosis, or malignant neoplasm. Summary statistics are provided as medians with Q1, Q3 or numbers with percentages as appropriate. Abbreviations: NIV, Non-invasive ventilation; HFO, high-flow oxygen; CCI, Charlson comorbidity index; NRL, neutrophil-to-lymphocyte ratio; CR, Chest radiography.

**Table S-2. Sensitivity analysis of the propensity score with a caliper width of 0.2 of the standard deviation of the logit.**

|  |  | **Before Propensity Score Matching** | | |  | **After Propensity Score Matching** | | | | |
| --- | --- | --- | --- | --- | --- | --- | --- | --- | --- | --- |
|  |  |  |  |  |  | **Tocilizumab + Baricitinib** | **Tocilizumab** |  | **Overall mortality 28-day**  **Hazard ratio (95% CI)*** | |
| **Variable** |  | **All** | **Tocilizumab + Baricitinib** | **Tocilizumab** |  |  |  |  | **Unadjusted** | **Adjusted** |
| **Patients, no.** |  | **994** | **110** | **884** |  | **41** | **41** |  | - | - |
| **Male sex** |  | **636 (64)** | 74 (67) | 562 (64) |  | 25 (61) | 25 (61) |  | 1.61 (0.50-5.22) | 3.89 (0.63-23.99) |
| **Age, years** |  | **66 (54, 76)** | 72 (60, 79) | 65 (54, 76) |  | 70 (58,77) | 70 (61, 77) |  | 1.19 (1.09-1.30) | **1.17 (1.05-1.32)** |
| **CCI, points**  **Any comorbidity***  **Cardiovascular disease**  **Hypertension**  **Diabetes**  **Chronic obstructive lung disease** |  | **3 (1, 4)**  **735 (74)**  **311 (31)**  **465 (47)**  **236 (24)**  **54 (5)** | 3 (2, 5)  99 (90)  47 (43)  64 (58)  35 (32)  12 (11) | 2 (1, 4)  636 (72)  264 (30)  401 (45)  201 (23)  42 (5) |  | 3 (2, 5)  33 (81)  16 (39)  20 (49)  13 (32)  4 (10) | 3 (2, 5)  33 (81)  22 (54)  26 (63)  14 (34)  1 (2) |  | **1.44 (1.25-1.65)**  -  -  -  -  - | 1.18 (0.86-1.62)  -  -  -  -  - |
| **WHO severity score**  **SOFA score**  **Peak FIO2**  **Remdesivir use**  **Baricitinib use** |  | **4 (4, 4)**  **2 (1, 3)**  **36 (32, 50)**  **713 (72)**  **110 (11)** | 4 (4, 6)  3 (2, 4)  100 (100, 100)  86 (78)  110 (100) | 4 (4, 4)  2 (1, 2)  36 (32, 50)  627 (71)  0 (0) |  | 4 (4, 6)  3 (2, 4)  100 (79, 100)  31 (76)  41 (100) | 4 (4, 5)  3 (2, 3)  95 (90, 100)  26 (63)  0 (0) |  | 1.05 (0.56-1.98)  -  **1.04 (1.02-1.07)^#^**  0.40 (0.15-1.23)  0.45 (0.15-1.33) | 1.23 (0.39-3.89)  -  1.03 (1.01-1.06)^#^  1.26 (0.14-11.42)  1.04 (0.23-4.66) |
| **eGFR, ml/min**  **eGFR ≤ 30 ml/min**  **CRP, mg/L**  **IL-6, pg/mL**  **D-dimer, mcg/mL**  **Bilateral lung infiltrates**  **Length of hospital stay, days** |  | **89 (69, 103)**  **50 (5)**  **61 (20, 118)**  **180 (74, 447)**  **1.0 (0.6, 2.0)**  **882 (89)**  **6 (4, 11)** | 83 (55, 98)  9 (8)  33 (6, 104)  196 (84, 485)  1.1 (0.6, 2.1)  107 (97)  19 (13, 27) | 90 (73, 104)  41 (5)  81 (50, 128)  167 (44, 439)  0.9 (0.5, 1.8)  775 (88)  6 (4, 9) |  | 84 (56, 98)  1 (2)  33 (5, 104)  211 (66, 487)  1.1 (0.6, 2.5)  39 (95)  17 (13, 23) | 73 (53, 88)  3 (7)  95 (51,164)  170 (38, 513)  0.8 (0.5, 1.5)  36 (88)  13 (8, 24) |  | **-**  -  -  -  -  -  - | -  -  -  -  -  -  - |
| **Clinical events at 28 days**  **Overall mortality**  **ICU admission**  **Mechanical ventilation**  **Documented infection**  **Thromboembolic events**  **Overall mortality at 60 days**  **Overall mortality at 90 days** |  | **74 (7)**  **93 (9)**  **75 (8)**  **49 (5)**  **48 (5)**  **90 (9)**  **91 (9)** | 28 (26)  42 (38)  34 (31)  19 (17)  21 (21)  33 (30)  33 (30) | 46 (5)  51 (6)  41 (5)  30 (3)  27 (3)  57 (6)  58 (7) |  | 6 (15)  17 (41)  13 (32)  6 (15)  3 (7)  9 (22)  9 (22) | 7 (17)  11 (27)  10 (24)  5 (12)  0 (0)  9 (22)  9 (22) |  | -  -  -  -  -  -  - | -  -  -  -  -  -  - |

* Hazard ratio estimates shown are those of the variables included in the adjusted model. ^#^Hazard ratio for time-varying fraction of inspired oxygen. Summary statistics are provided as medians with Q1, Q3 or numbers with percentages as appropriate. Abbreviations: CI, confidence interval; CCI, Charlson comorbidity index; WHO, World Health Organization; SOFA, Sequential Organ Failure Assessment; FiO2, fraction of inspired oxygen; eGFR, Chronic Kidney Disease Epidemiology Collaboration (CKD-EPI) estimation of glomerular filtration rate; CRP, C-reactive protein; IL, Interleukin; NIV, Non-invasive respiratory support; HFO, high-flow nasal oxygen; ICU, Intensive Care Unit.

**Table S-3. List of secondary co-infections in study patients by treatment group.**

|  | **Tocilizumab + Baricitinib** | **Tocilizumab** |
| --- | --- | --- |
| **N (%)**  **Bacterial respiratory coinfection***  **Bacteremia^#^**  **Invasive fungal infection^&^**  **Non-respiratory sepsis^¥^**  **Other^§^** | **17 (17.9)**  **6**  **5**  **4**  **1**  **1** | **10 (10.5)**  **5**  **2**  **0**  **2**  **1** |

*, In tocilizumab plus baricitinib group: *Staphylococcus aureus* (n=1), *Pseudomonas aeruginosa* (n=2), *Serratia marcescens* (n=1), infection suspected based on clinical ground (n=2); in tocilizumab group: *Pseudomonas aeruginosa* (n=1); infection suspected based on clinical ground (n=4); ^#^, In tocilizumab plus baricitinib group: *Pseudomonas aeruginosa* (n=2), *Stenotrophomonas maltophilia* (n=1), *Enterococcus spp.* (n=2); in tocilizumab group: *Pseudomonas aeruginosa* (n=1), *Staphylococcus aureus* (n=1). ^&^, *Aspergillus spp* (n=3), *Candida spp* (n=1). ^¥^, In tocilizumab plus baricitinib group: Abdominal sepsis; in tocilizumab group: unknown origin (n=1), urinary tract (n=1); ^§^, Acute hepatitis C virus infection (n=1) in tocilizumab plus baricitinib group and tuberculosis in tocilizumab group.

**Figure S-2. Adjusted Cox regression model hazard ratios for the combination of tocilizumab plus baricitinib versus tocilizumab alone for thromboembolic events.**

**
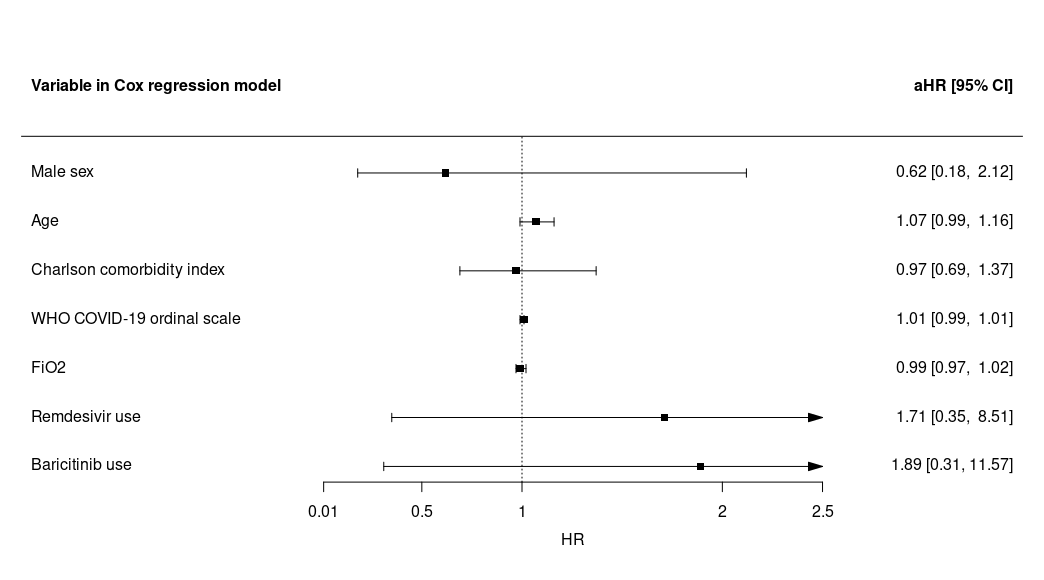
**

Abbreviations: aHR, adjusted hazard ratio; WHO, World Health Organization; FiO2, time-varying fraction of inspired oxygen.

**Figure S-3. Predictors for overall 28-day mortality in multivariate Cox regression model in subjects** **receiving tocilizumab.**

**
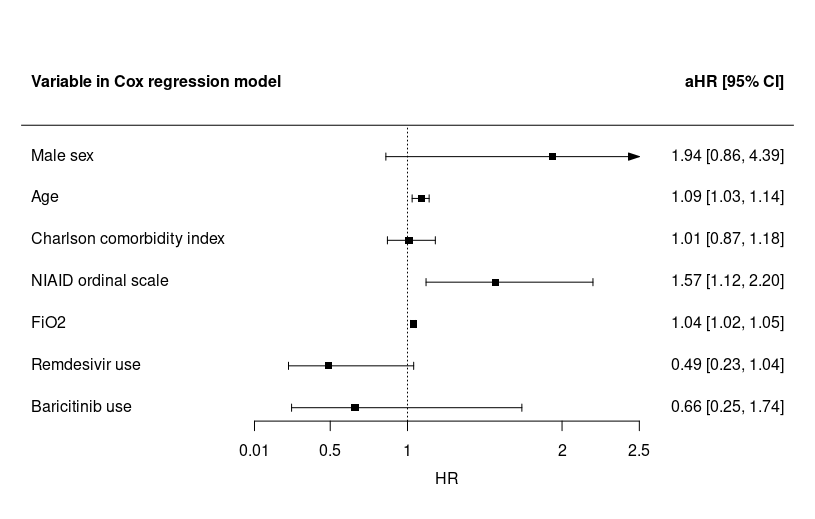
** Abbreviations: aHR, adjusted hazard ratio; NIAID, National Institute of Allergy and Infectious Diseases (see: “Beigel JH, Tomashek KM, Dodd LE, et al. Remdesivir for the treatment of Covid-19-Final Report. NEJM 2020 Oct 8” for NIAID ordinal scale details); FiO2, time-varying fraction of inspired oxygen.
